# Neoadjuvant followed by adjuvant pembrolizumab in melanoma: time biases in the data analysis of the SWOG S1801 trial.

**DOI:** 10.1016/j.tranon.2024.101959

**Published:** 2024-04-14

**Authors:** Timothée Olivier, Vinay Prasad

**Affiliations:** a Department of Oncology, Geneva University Hospital, 4 Gabrielle-Perret-Gentil Street, Geneva 1205, Switzerland; b Department of Epidemiology and Biostatistics, University of California San Francisco, 550 16th St, 2nd Fl, San Francisco, CA 94158, USA

**Keywords:** Melanoma, Neoadjuvant, Immunotherapy, Informative censoring

## Abstract

The SWOG S1801 trial investigated the role of pembrolizumab, an anti-PD1 immune checkpoint inhibitor, in the perioperative setting of stage III or IV melanoma. This phase 2 trial compared two groups: one receiving pembrolizumab both before and after surgery (neoadjuvant-adjuvant), and another receiving it only post-surgery (adjuvant-only), with event-free survival (EFS) as the primary endpoint. Neoadjuvant strategies, involving pre-surgical drug administration, potentially offer rapid tumor control and a unique opportunity to assess tumor response. However, they may expose to toxicity and delay or preclude surgery. The study met its primary endpoint, with a 72 % EFS rate in the neoadjuvant–adjuvant group, and 49 % in the adjuvant group. Here, we question the results’ applicability with three potential limitations. Key concerns include an arbitrary rule in event assignment, possibly affecting the event distribution over time. Second, different rates of early censoring between groups introduce the possibility of informative censoring, which could have led to an artefactual benefit in EFS. Lastly, phase 2 trial results, by definition, carry risk of fluke results, and should be confirmed in phase 3 trial before wide adoption. Collectively, these factors must be integrated into a careful interpretation of the SWOG S1801 trial outcomes. More robust data are needed to fully appraise strengths and limitations of neoadjuvant pembrolizumab in melanoma treatment.

## Highlights

• Neoadjuvant pembrolizumab in resectable melanoma was studied in the S1801 trial.
• The study showed an improvement in event-free survival in the neoadjuvant group.
• High and imbalanced rates of early censoring could have been informative.
• An unusual and arbitrary rule affected the event distribution over time.
• The S1801 strategy should be confirmed in a phase 3 trial before changing practice.

## Graphical abstract

**Figure:**
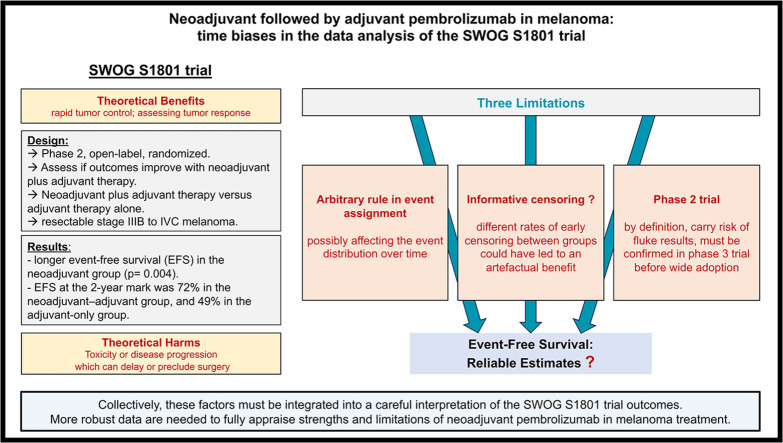

## Introduction

Neoadjuvant immunotherapy strategies have gained recent attention. Since 2021, three anti-PD1 monoclonal antibodies have been approved by the US Food and Drug Administration (FDA) in the neoadjuvant setting of solid tumors: pembrolizumab in triple-negative breast cancer based on the KEYNOTE-522 trial [1], nivolumab and pembrolizumab in non-small cell lung cancer (NSCLC) based on the CheckMate-816 trial [2], and the KEYNOTE-671 trial [3], respectively.

In patients with resectable stage III or IV melanoma, the SWOG S1801 cooperative study investigated if pembrolizumab would be more effective if given both before and after surgery (neoadjuvant followed by adjuvant therapy), as compared with strategies delivering pembrolizumab only as a postoperative treatment (adjuvant) [4]. The results were positive, with a prolonged event-free survival (EFS) in the neoadjuvant group. Here, we present three significant limitations about the SWOG S1801 results which limit their potential to inform practice. The first limitation is an unexplained rule (assigning some events on day 84) which obfuscates the distribution of events over time. The second limitation pertains to higher rates of censoring in the neoadjuvant group at early time points, raising the possibility for informative censoring. Lastly, phase 2 trials may lead to spurious results due to lack of power, which is the reason why such results must be recapitulated in phase 3 trials before being adopted.

## The SWOG S1801 trial

The study was a phase 2 trial enrolling patients with surgically resectable, clinically detectable stage IIIB to IVC melanoma. They were randomized to two groups: the neoadjuvant-adjuvant group received a regimen of three doses of preoperative pembrolizumab, followed by surgical resection, followed by 15 postoperative pembrolizumab doses. Alternatively, the “adjuvant-only” group underwent surgery followed by pembrolizumab treatment, administered intravenously every 3 weeks (totaling 18 doses). The primary outcome was event-free survival in the intention-to-treat population, with events defined as “disease progression or toxic effects that precluded surgery; the inability to resect all gross disease; disease progression, surgical complications, or toxic effects of treatment that precluded the initiation of adjuvant therapy within 84 days after surgery; recurrence of melanoma after surgery; or death from any cause.”.

After a median follow-up period of 14.7 months, it was observed that the neoadjuvant–adjuvant group (comprising 154 patients) demonstrated a significantly longer event-free survival as compared to the adjuvant-only group (comprising 159 patients) (*p* = 0.004). The event-free survival at the 2-year mark was 72 % (95 % CI, 64 - 80) in the neoadjuvant–adjuvant group, and 49 % (95 % CI, 41 - 59) in the adjuvant-only group.

The positive results of this phase 2 trial led some to advocate for a change in practice [5]. We agree neoadjuvant approaches may have appealing features, both practically (allowing to initiate a treatment more rapidly than a surgery, and allowing the surgery to be easier, for instance) and biologically (efficacy from checkpoint inhibitors could be greater when tumor has not yet been removed, due to immunogenicity rationale [6,7]). However, there are also potential downsides, one of them being side effects from the neoadjuvant position potentially precluding future surgery.

Nonetheless a captivating rationale, it must pass through the sieve of evidence-based evaluation before gaining wide acceptance. This step is critical to prevent being misled by false signals, which could lead to practices being established and later overturned, a term coined medical reversal [8].

## Disease progression or adverse events may delay or preclude surgery

A key concern with neoadjuvant strategies as compared with surgery-first approaches is whether the surgery could delay or even cancel surgery. In the S1801 trial, it is reported that 16 patients randomized to the neoadjuvant strategy (out of 134, 10.3 %) did not undergo surgery [4]. There are various reported explanations with adverse events in 1 patient, disease progression in 12, and withdrawal of consent of 2 additional patients.

This issue is not unique to melanoma. In a systematic review from neoadjuvant approaches in NSCLC, the authors found a wide range of surgery cancellation rates (from 0 % to 45.8 %), with adverse events being the less frequent explanation for cancelation, and disease progression being a common reason [9]. Conversely, adverse events were the leading cause for delays of surgery, which occurred from 0 to 31.3 % across selected trials [9]. However, it is unclear to which extent the data observed in patients with NSCLC may apply to patients with melanoma, as the disease and the surgical procedures are differing between both tumor types.

In patients with macroscopic palpable stage III melanoma, within the OpACIN phase 1b trial, all 10 patients enrolled in the neoadjuvant group (2 cycles of ipilimumab plus nivolumab) underwent surgery as planned [10]. The OpACIN-neo phase 2 trial investigated 3 different doses or schedules of dual checkpoint inhibition (ipilimumab and nivolumab) in the neoadjuvant setting of patients with stage III melanoma [11]. Of the 86 patients that initiated therapy, 85 ultimately underwent surgery . However, three patients had surgical delays due to adverse events (3.5 %), and one additional patient had the surgery cancelled because of an adverse event (1.2 %).

The differences seen between OPACIN and OpACIN-neodata, using dual checkpoint inhibition strategies, and those from the SWOG S1801 trial, which used anti-PD1 monotherapy, known to be less toxic, may also be explained by differences in patient selection between trials, which might have influenced the risk of disease progression [12].

## Time biases: guarantee-time bias and a novel “shorter-time bias”

**Fig. 1:**
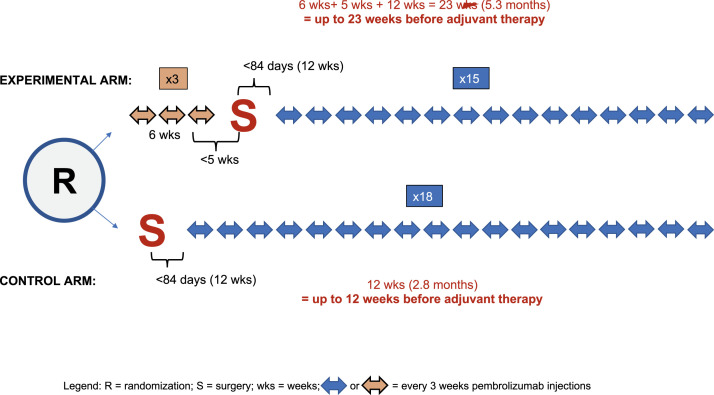
Study Design of the SWOG1801 trial, With Time Periods Before The Initiation of the Adjuvant Portion of Therapy in Both Arms, According To The Protocol.

In Fig. 1, we illustrate the SWOG S1801 study design with the different time-periods allocated in each treatment arm. Patients randomized to “surgery-first” (control) could have up to 84 days (12 weeks) before initiating adjuvant pembrolizumab. Conversely, patients allocated to the “neoadjuvant-first” strategy started with 3 cycles of pembrolizumab, equaling 6 weeks until receiving the third dose; followed by surgery which should occur no later than 5 weeks after the last pembrolizumab dose. Post-surgery, similarly to control arm patients, patients could wait up to 12 weeks before initiating the adjuvant portion. In other words, events happening before the adjuvant portion could occur up to 23 weeks in the neoadjuvant arm, and up to 12 weeks in the adjuvant-only arm – almost half shorter.

A very unusual rule was found in the protocol, which resulted in masking the distribution of early events over time, yet being a core principle in Kaplan-Meier analyses. The rule is the following: “On both arms, all participants who do not register for adjuvant therapy (for whatever reason) will be assigned the event time of 84 days.” (Protocol version Version Date 2/11/2022, Page 52). [4]

There is no scientific justification found for this rule within the protocol. This rule is explaining the unusual shape of the Kaplan-Meier curve, displaying a vertical drop at 84 days. Not only this design is introducing a “guarantee-time bias”, meaning that patients with early events will be attributed a later date (84 days = 12 weeks) for no reason, but this is also introducing a novel type of bias: attributing an earlier event date in cases of patients having the event after 12 weeks, which could happen in the neoadjuvant arm (up to 23 weeks).

One could argue this rule could have only favored the neoadjuvant arm. This is incorrect simply because we have no idea of the real distribution of the events currently attributed to the day 84: they could have varied greatly in both arms.

## Informative censoring

Based on the reconstructed individual patient data (IPD), we estimated 17.5 % of patients (*n* = 27) were censored during the first 6 months in the experimental group, as compared with 11.9 % in the control arm (*n* = 19) [13].

To provide some perspective, an empirical analysis led by Rosen et al. found that the weighted average of difference in PFS censoring events between arms at the first time-point was 2 point-percentage, with more censoring occurring in the control arm [14]. In the S1801 trial, we estimated a 5.6 point-percentage difference with more patients censored in the experimental therapy . Such imbalance in rates of early censoring raise concerns about the presence of informative censoring. In other words, censoring events could have not occurred “at random”, but due to reasons related to the allocated arm. Any withdrawal from the protocol before tumor assessment, for instance due to toxicity, can lead to censoring if the time between the end of therapy and the next tumoral assessment is too long. As such, it is unsurprising to see higher rates of censoring in the neoadjuvant arm, where toxicity from systemic treatment can arise earlier than in the adjuvant only arm. Such imbalance in censoring rates due to toxicity may preferentially censor the frailer patients, which could be those more likely to experience an event.

**Fig. 2:**
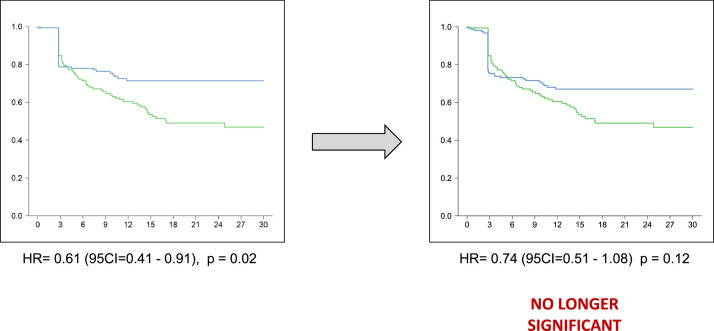
Reconstruction Of Kaplan-Meier Curves Assuming Every Patients Censored In Excess In The Experimental Arm During The First Time-Interval (*n* = 8) Had An Event Instead Of Being Censored.

We conducted a conservative sensitivity analysis, in which patients censored in excess in the experimental arm during the first 6 months (*n* = 8) would have experienced an event instead of being censored. After reconstructing synthetic IPD from the published Kaplan-Meier analysis, we randomly selected 8 censored patients and modified their status as experiencing the event at the time of censoring: the statistical significance is no longer found (Fig. 2).

This sensitivity analysis demonstrates, by simulating changes in the fate of a fraction of censored patients over the first time-interval, that the SWOG S1801 results are vulnerable to informative censoring. The abstracted data and code for re-analysis are openly available (github.com/TimotheeMD/SWOG1801_reanalysis).

## Underpowered phase 2 trial

The EFS results observed in the SWOG 1801 trial are those of a phase 2 trial. Underpowered phase 2 trials may not only miss a true effect, but also have an increased risk of findings spurious positive results [15]. The case of olaratumab in sarcoma serves as a notable example. Olaratumab, a monoclonal antibody targeting PDGFR-α, was tested in patients with metastatic sarcomas, in addition to doxorubicin. A phase 2 clinical trial showed a 12 months overall survival gain with the novel therapy, leading to its accelerated approval [16]. However, the confirmatory phase 3 trial – the ANNOUNCE trial – showed absolutely no benefit between arms, leading the drug to be pulled-out off the market [17].

Although phase 3 trials investigating the role of neoadjuvant immunotherapy in melanoma are ongoing, we are not aware of a phase 3 trial aiming at confirming the results of the SWOG S1801 strategy. The phase 3 NADINA trial is investigating the role of 2 cycles of ipilimumab (80 mg) and nivolumab (240 mg) before surgery [18]. Other neoadjuvant immunotherapy strategies, like intratumoral injections, are being investigated within ongoing or completed phase 3 trials (NeoDREAM NCT03567889, PIVOTAL NCT02938299) [19].

## Conclusion

Enthusiasm about neoadjuvant-first strategies in melanoma are grounded into biological data (potential for enhanced immune response when the tumor is within the body) and the potential for initiating a treatment rapidly. The main downsides are side effects or tumor progression potentially precluding or delaying surgery. The SWOG S1801 addressed this interesting research question. However, a very unusual rule masked the distribution of events over the first months in both arms. In addition to higher rates of early censoring events in the neoadjuvant arm, and with the inherent statistical limitation of a phase 2 trial, those factors hamper the ability for the S1801 trial to inform current practice.

## Funding

This project was funded by Arnold Ventures, LLC through a grant paid to the University of California, San Francisco.

## CRediT authorship contribution statement

**Timothée Olivier:** Methodology, Conceptualization, Software, Visualization, Writing – original draft, Writing – review & editing. **Vinay Prasad:** Validation, Writing – review & editing.

## Declaration of competing interest

Dr Vinay Prasad reported receiving research funding from Arnold Ventures LLC through a grant made to UCSF; royalties for books and writing from Johns Hopkins Press, MedPage, and the Free Press; and consulting fees from UnitedHealthcare and OptumRX. He also reported receiving revenue from Patreon, YouTube, and Substack for the podcasts Plenary Session, VPZD, and Sensible Medicine; for the newsletters Sensible Medicine, The Drug Development Letter, and VP's Observations and Thoughts; and for the YouTube channel Vinay Prasad MD MPH. Dr Timothée Olivier has no conflicts of interest to declare.
